# Habits Related to Strength Training of Brazilian Recreational Runners

**DOI:** 10.3390/sports13010003

**Published:** 2024-12-25

**Authors:** Wagner K. A. Santos, Lucas D. M. Forte, Alexandre S. Silva, Hallisson V. de O. Rufino, Lucas de F. Vieira, João M. F. Lima Silva, Mabliny Thuany, Reginaldo Gonçalves, Ytalo M. Soares

**Affiliations:** 1 Training and Sports Performance Study Group, Department of Physical Education, Federal University of Paraiba, João Pessoa 58051-900, Brazil; kayse_wagner@hotmail.com (W.K.A.S.); lucas.dmf@hotmail.com (L.D.M.F.); alexandresergiosilva@yahoo.com.br (A.S.S.); hallissonvinicius@hotmail.com (H.V.d.O.R.); llukas_freitas@hotmail.com (L.d.F.V.); joaomarcosef@gmail.com (J.M.F.L.S.); 2 Department of Sport, State University of Pará, Belém 66050-540, Brazil; mablinysantos@gmail.com; 3 Load Evaluation Laboratory, School of Physical Education, Physiotherapy, and Occupational Therapy, Federal University of Minas Gerais, Belo Horizonte 31270-901, Brazil; reginaldociclismo@hotmail.com

**Keywords:** endurance, resistance training, strength training, training practices, recreational runners

## Abstract

Despite strength training (ST) being well characterized by professional runners, little is known about the inclusion of ST models for recreational runners. Thus, the present study aimed to investigate the presence of ST in the training practices of recreational runners, with a focus on understanding its characteristics and the motivations of recreational athletes for including ST in their routines. To this end, 801 recreational runners (493 male and 308 female) completed a structured questionnaire regarding their training habits, the inclusion of ST, and its characteristics, concerning the type of ST, training volume, and the reasons for including ST in their training programs. To assess the possible associations between categorical variables, data were analyzed using the chi-square test for independent samples. Approximately 625 runners (78.1%) reported that they included ST in their training routine, with a statistically significant difference between the sexes (men: 73.5% vs. women: 85.4%; X^2^ = 14.09; *p* = 0.01). Traditional strength training (TST) was the predominant type of ST included (78.5%), with most participants performing 2–4 sets and 8–12 repetitions per set at a frequency of 3–4 sessions per week. The importance of ST was primarily attributed to performance improvement (85%). The results suggest that recreational runners incorporate different types of ST into their training routines, with TST being the predominant type perceived by runners as a valuable asset for improving running performance.

## 1. Introduction

In recent decades, endurance running has gained significant popularity among non-professional runners, becoming one of the most commonly practiced sports during leisure time [1]. At the same time, interest in understanding contemporary trends and the specific demands of this subgroup of runners has grown [2,3,4]. Typically, recreational runners aim to improve their physical capacity and performance through structured training programs [5,6]. However, much of the scientific knowledge on sports training is based on studies with elite athletes, underscoring the need for greater focus on recreational runners.

In addition to running-specific training, strength training (ST) has been identified by sports and exercise scientists as an effective strategy for enhancing running economy and performance in middle- and long-distance runners [7,8] while also reducing injury risk [9,10,11,12,13,14,15,16]. ST can be defined as the systematic and organized use of exercises in which the body, through muscular actions, works against a certain resistance with the goal of maintaining or optimizing muscular strength. To achieve this, various means and methods can be employed, ranging from the traditional use of weights and machines to other types of resistance, including exercises that utilize the body’s own weight. For instance, García-Pinillos et al. [17] examined the relationship between ST habits and performance levels among amateur runners, finding that those with higher performance levels were less likely to exclude ST from their training routines. Furthermore, ST was typically performed at light to moderate intensities (21.5% to 52.2% of 1RM) with high volume (6–12 to 13–20 repetitions) by the majority of athletes.

Despite the potential of ST to induce neuromuscular adaptations—such as optimizing muscle elasticity and increasing muscle–tendon stiffness—and mechanical improvements, such as reducing energy cost through enhanced stride frequency, stance time, and stride length [8,18,19,20,21], many recreational runners still do not incorporate ST into their training. Blagrove et al. [5] reported that 23.1% of 667 middle- and long-distance runners did not include ST in their routines. Similarly, a study on the training habits of 93 Olympic marathon runners (elite and national level) found that nearly half of the participants did not perform ST throughout the year [22].

Research on the training habits of recreational runners, particularly regarding ST, remains scarce. Understanding how recreational runners integrate ST into their routines could assist coaches and scientists in developing optimal training prescriptions and improving performance outcomes. Additionally, this knowledge is crucial for fostering dialogue among coaches about training strategies, improving professional practices, and raising awareness among recreational runners about the benefits of ST for performance enhancement.

Therefore, this study aims to examine the presence of ST in the training practices of recreational runners, with a focus on understanding its characteristics and the motivations of recreational athletes for including ST in their routines. Based on the authors’ theoretical and practical experiences, we hypothesize that there is a high prevalence of ST among recreational runners, especially in the form of machine-based exercises and free weights (traditional strength training—TST), following classical non-athlete training prescriptions.

## 2. Methods

### 2.1. Ethical Aspects

Participants were informed of their voluntary participation and their right to decline answering specific items. All participants received detailed information about the study’s objectives and scope and provided informed consent before participation. The study was approved by the local ethics committee (protocol number: 4.682.96).

### 2.2. Study Design and Sample Characteristics

This cross-sectional, internet-based study included a sample of 801 recreational runners of both sexes (308 women and 493 men). The participants had a mean age of 39.05 ± 9.63 years, a mean height of 170 ± 0.91 cm, and a mean body mass of 74.53 ± 13.64 kg. The participants were recruited from the nine capitals of northeastern Brazil. Eligibility criteria included being over 18 years old, having a minimum of 12 months of running experience, training regularly for at least the six months prior to data collection, and running at least twice a week with a minimum weekly volume of 20 km. Participants who did not meet these criteria, failed to complete all questionnaire items, or submitted duplicate responses were excluded from the study.

### 2.3. Questionnaire and Data Collection Procedures

The questionnaire “Training Habits of Runners in the Capitals of Northeastern Brazil” was developed specifically for this study. Before its application, the questionnaire underwent a content validation process [23,24,25].

#### 2.3.1. Questionnaire Content Validation Process

The expert panel consisted of two groups: seven physical education academics with doctoral degrees and seven running coaches. These experts were asked to independently evaluate the questionnaire items. The panel’s role was to assess each item based on three criteria: clarity of language, theoretical relevance, and alignment with the study objectives. These criteria were adopted to ensure the appropriateness of the questions and to modify or eliminate any items that were ambiguous, unclear, duplicative, or used jargon with subjective interpretations [23,24]. Due to their familiarity with the technical terms used in running, the coaches only assessed the items for clarity of language. Both groups used a Likert-type scale to rate each item from 1 to 4, where 1 indicated the item did not meet the criterion at all and 4 indicated that it fully met the criterion.

After the expert evaluation, the Content Validity Index (CVI) was calculated for each item (CVIi) and for the overall questionnaire (CVIt), as proposed by Polit and Beck [24,26]. The CVIi is the proportion of experts who rated the item as “3” or “4”, divided by the total number of experts. The CVIt represents the average of all item CVIs, divided by the total number of items, considering clarity of language, practical relevance, and theoretical relevance. A minimum CVI of 0.78 was deemed acceptable for both individual items and the overall instrument [25].

The final version of the questionnaire was composed of a total of 34 questions, with both open-ended and closed-ended questions divided into four domains: demographic information (i.e., age, gender, capital of residence, and education); technical information of the last six months (running practice time and their personal best time in a 5 km trial); characteristics of running training of the last six months (frequency and weekly training volume) and the characteristics of strength training (type, timing of ST sessions, and range of repetition per set performed). In the present study, strength training was considered as resistance exercises with one’s body weight, with machines or free weights, or with any specific resource that can increase the resistance of the exercise.

#### 2.3.2. Procedures for Data Collection

The questionnaire was transcribed for an online platform (Google Forms) and sent to runners through a link. The strategy used to spread the instrument was to contact groups of runners and clubs. It was also disseminated through social media platforms (e.g., Instagram and WhatsApp 2.22.8).

To avoid potential selection bias based on strength training habits, participants were invited to respond about their general training routines, including their running habits, without an emphasis on strength training initially. Questions related to strength training practices were only introduced later in the questionnaire, beginning at question 19, in the final section of the survey. This design allowed all participants—whether they practiced strength training or not—to complete the main portion of the survey, including all questions on running and general training habits. By the time they reached the strength training section, participants who did not engage in strength training simply responded accordingly, without any need to discontinue the survey.

### 2.4. Statistical Analysis

Frequency analysis, percentages, and descriptive statistics (mean and standard deviation) were used to summarize the data. The chi-square (χ^2^) test was applied to compare differences in proportions. Data were analyzed using IBM SPSS 20.0 with statistical significance set at *p* < 0.05.

## 3. Results

The participants’ characteristics, including sex, age, height, body mass, and weekly running training volume, are presented in Table 1. When analyzed by sex, men reported an average of 6.2 ± 6.47 years of running experience, while women reported 4.0 ± 3.73 years; in terms of their best 5 km times, men recorded an average of 25.3 ± 5.58 min, whereas women averaged 29.6 ± 5.37 min. Among the men, 20.48% covered up to 15 km per week; 24.34% covered between 15 and 25 km; 24.35% covered between 25 and 35 km; and 30.83% covered more than 35 km. Regarding the women, 23.37% covered up to 15 km per week; 38.31% covered between 15 and 25 km; 19.8% covered between 25 and 35 km; and 18.5% covered more than 35 km per week. In terms of frequency, men ran 3.81 ± 1.20 times per week, while women ran 3.37 ± 0.73 times per week. Additionally, 602 participants (75.0%) reported training with running groups.

### 3.1. Strength Training Characteristics

A total of 625 runners (78.1%) reported including ST in their training routines. Comparisons revealed significant differences between sexes (χ^2^ = 14.09, *p* = 0.01), with men reporting higher participation in ST (58.1%, *n* = 362) compared to women (41.9%, *n* = 263). Among the types of ST performed, TST was the most commonly reported (78.5%, *n* = 491), followed by bodyweight exercises (49.4%, *n* = 309) and functional training (21.1%, *n* = 130) (Table 2).

A smaller proportion of runners engaged in plyometric exercises (14.4%, *n* = 90), uphill running (11.8%, *n* = 74), Pilates (4.6%, *n* = 29), and resistance band training (2.9%, *n* = 18). Among participants who included ST in their routines, approximately half (47.2%) performed at least one type of ST, 45.9% reported engaging in two or three types, and a small percentage (6.9%) practiced at least four different types of ST. Only 5.5% of men and 4.6% of women did not perform strength training for the lower limbs.

Table 3 provides details on the ST characteristics of the runners. In terms of repetitions per set, only 8.8% of runners performed sets with 4–6 repetitions and 6.1% performed sets with more than 15 repetitions. A total of 32.4% completed sets of 1–3 repetitions, while the majority (46.3%) performed 8–12 repetitions per set.

### 3.2. Motive and Importance Level of ST

The primary reasons for including strength ST in runners’ routines are presented in Table 4. Among those who incorporated ST, 85% considered it very important for improving performance. The most commonly cited reason for ST inclusion among both sexes was “reducing the risk of injury” (78.1%, *n* = 488), followed by “improving running performance” (72.2%, *n* = 451). A significantly greater proportion of women compared to men cited “improving aesthetics” (χ^2^ = 10.1, *p* = 0.001), “improving running technique” (χ^2^ = 5.03, *p* = 0.025), and “improving health” (χ^2^ = 11.7, *p* = 0.001) as key reasons for including ST in their training.

When asked why they did not include ST in their routine, 176 participants (21.9%) indicated they performed no form of strength training. The most frequently reported reason was a “lack of time” (58.1%). Additionally, a significant difference was found between men and women regarding the reason “lack of knowledge”, with a greater proportion of men (20.8%) compared to women (7.5%) citing this as a factor preventing them from including ST (χ^2^ = 3.86, *p* = 0.049).

## 4. Discussion

The objective of this study was to investigate the presence of ST among recreational runners, examining its characteristics and the motivations of recreational athletes for including ST in their routines. The findings reveal that a majority of recreational runners (78.1%) included ST in their routines, with resistance training being the most commonly adopted form. The main motivators for incorporating ST were injury prevention (78.1%) and performance improvement (72.2%). Among the 21.9% who did not include ST in their training, the primary reason cited was “lack of time”.

The frequency of ST reported in this study aligns with previous research by Blagrove et al. [5], Loudonet et al. [27], and Griffinet et al. [28], which found a prevalence of ST among runners (76.9%, 77.9%, and 88.0%, respectively). However, those studies focused on competitive athletes, while our study involved recreational runners. A higher prevalence of ST (91.6%) was reported by Garcia-Pinillos et al. [17] among amateur runners, suggesting that ST is a common auxiliary training method regardless of competitive level.

No significant differences were found between men and women regarding the importance of ST for performance, with 84.8% of men and 87.5% of women considering it highly important (*p* = 0.072). This highlights the relevance of ST for endurance sports across various runner profiles.

As hypothesized, TST was the most common form of ST (78.5%). Although literature suggests that plyometric training is effective for performance improvement [12,29,30], only a small percentage (14.4%) of runners in this study included plyometric exercises. This contrasts with other studies, such as those of Griffin et al. [28] and Garcia-Pinillos et al. [17], where plyometric training had a higher prevalence (35.4% and 46.8%, respectively). This suggests that recreational runners and their coaches might lack knowledge about the most effective types of ST for performance enhancement. Cultural practices and differences in training background could also play a role in exercise selection.

Given that TST is the most common form of ST used by runners, it is crucial to examine aspects such as frequency, volume, and intensity. In this study, half of the participants performed TST three to four times per week, whereas previous studies suggest that two to three weekly sessions are sufficient to enhance long-distance running performance [10,18]. Regarding volume, most runners performed two to four sets per exercise, with the majority executing 8–12 repetitions per set, which aligns with the literature for endurance runners [10].

Injury prevention was the most cited reason for including ST, corroborating previous research on injury reduction through ST across sports [31,32,33]. Additionally, 72.2% of participants stated that improving performance was a key motivation for ST inclusion, a higher proportion than the 53.8% reported in a study by Blagrove et al. [5] which focused on competitive athletes.

Conversely, 21.9% of participants did not perform any ST. This is higher than the less than 10% non-participation reported by Garcia-Pinillos et al. [17] and the 23.1% found by Blagrove et al. [5]. The main barrier identified in this study was a “lack of time”, similar to previous findings among endurance athletes [34]. A significant gender difference was observed, with men (20.8%) more likely than women (7.5%) to cite “lack of knowledge” as a reason for not performing ST (*p* = 0.049).

Although previous research has mentioned the benefits of ST for endurance athletes [14,35], there is a limited understanding of the barriers and misconceptions recreational runners face. Factors such as “lack of time”, “lack of gym access”, and insufficient knowledge about exercise types and techniques are common obstacles [36]. Future studies should further explore the knowledge gaps and factors influencing ST adoption among recreational runners to optimize their training programs.

A limitation of this study is the lack of data on why runners who do not perform ST might still perceive its importance. Additionally, this study’s exploratory nature prevents causal inferences. More research is needed to explore the role of professional guidance in recreational runners’ ST practices and to determine the most effective ST regimens for performance improvement and injury prevention across different training levels.

## 5. Conclusions

The results of this study indicate that a majority of recreational runners incorporate various forms of ST into their training routines, with TST and bodyweight exercises being the most prevalent. Notably, half of the participants engage in three to four ST sessions per week, often utilizing a combination of two to three different types of ST. These findings suggest that coaches should familiarize themselves with diverse ST methods, their effective combinations, and the typical load distribution patterns throughout the training week for recreational runners.

The data further reveal that the primary motivations for runners to include ST are to minimize injury risks and enhance performance. Consequently, we advocate for the incorporation of ST—regardless of the specific type—focused on improving neuromuscular efficiency and ensuring strength transference to running performance.

Additionally, our findings highlight that a small percentage of recreational runners do not incorporate ST into their routines, primarily due to a lack of time or knowledge regarding the application of ST. Therefore, it is essential to educate recreational runners on the benefits of ST, including its role in reducing injury risks, enhancing running performance, and promoting overall health.

## Figures and Tables

**Table 1:** Participants’ characteristics.

	Male (*n* = 493)	Female (*n* = 308)	Total (*n* = 801)
	Mean ± SD	Mean ± SD	Mean ± SD
Height (cm)	175 ± 0.07	163 ± 0.07	170 ± 1.91
Body mass (kg)	81.2 ± 33.42	63.9 ± 8.87	74.63 ± 13.08
Age (y)	39.3 ±10.00	38.6 ± 8.87	39.05 ± 9.63
Average weekly running training	Freq. (%)	Freq. (%)	Freq. (%)
Up to 3 h	117 (14.6)	89 (11.1)	206 (25.7)
3–4 h	136 (17.0)	105 (13.1)	241 (30.1)
4–6 h	155 (19.4)	84 (10.5)	239 (29.8)
˃6 h	85 (10.6)	30 (3.7)	115 (14.4)
Inclusion of ST			
Yes	362 (73.5)	263 (85.4)	625 (78.1)
No	131 (26.5)	45 (14.6)	176 (21.9)

ST: strength training.

**Table 2:** Reported different types of exercises included in participants’ strength training program.

	Male (*n* = 362)	Female (*n* = 263)	Total (*n* = 625)	Sex Differences	
TYPE OF EXERCISE	Freq. (%)	Freq. (%)	Freq. (%)	X^2^	*p*
Traditional strength training	280 (57.0)	211(43.0)	491 (78.5)	0.750	0.386
Bodyweight	169 (54.7)	140 (45.3)	309 (49.4)	2.612	0.106
Functional training	75 (57.7)	55 (42.3)	130 (21.1)	0.003	0.953
Plyometric	51 (56.7)	39 (43.3)	90 (14.4)	0.068	0.795
Uphill runs	53 (71.6)	21 (28.4)	74 (11.8)	6.45	0.011 *
Pilates	14 (58.4)	15 (51.7)	29 (4.6)	1.16	0.281
Resisted elastic band	9 (50.0)	9 (50.0)	18 (2.9)	0.477	0.490

* Statistical difference.

**Table 3:** Characteristics of strength training.

	Male (*n* = 362)	Female (*n* = 263)	Total (*n* = 625)		
	Freq. (%)	Freq. (%)	Freq. (%)	*X* ^2^	*p*
PRACTICE EXPERIENCE OF ST					
Up to 1 year	118 (32.4)	91 (34.7)	209 (33.4)	9.181	0.010 *
1–3 year	121 (33.2)	59 (22.5)	180 (28.8)		
3+	123 (34.3)	112 (42.7)	235 (37.9)		
WEEKLY FREQUENCY					
Up to 2 sessions	149 (41.5)	77 (29.3)	226 (36.4)	11.634	0.003 *
3–4 sessions	171 (47.0)	139 (52.9)	310 (49.4)		
5+ sessions	42 (11.5)	47 (17.9)	89 (14.2)		
NUMBER OF SETS FOR EXERCISE					
1 to 2 sets	24 (6.6)	17 (6.5)	41 (6.5)	4.319	0.504
2 to 3 sets	156 (43.1)	106 (39.6)	262 (39.6)		
3 to 4 sets	141 (39.2)	109 (42.1)	250 (38.6)		
4 to 5 sets	23 (6.2)	24 (9.1)	47 (7.5)		
5+ sets	18 (4.9)	7 (2.7)	25 (2.9)		
REPETITION FOR SET					
1 to 3 reps	118 (32.4)	85 (32.3)	203 (32.4)	10.716	0.030 *
4 to 6 reps	43 (11.8)	12 (4.6)	55 (8.8)		
8 to 12 reps	157 (43.7)	131 (49.8)	288 (46.3)		
15+ reps	22 (6.0)	16 (6.1)	38 (6.1)		
Reps: repetitions					

* Statistical difference.

**Table 4:** Motive for runners to include ST, comparative between the genders.

VARIABLES	TOTAL (*n* = 625)		MEN (*n* = 362)		WOMEN (*n* = 263)		
		Frequency (%)					*p*-Value
Reduce the risk of injury	488	(78.1%)	278	(76.8%)	210	(79.8%)	0.362
Performance	451	(72.2%)	265	(73.2%)	186	(70.7%)	0.494
Improvement health	323	(51.7%)	166	(45.9%)	157	(59.7%)	0.001 *
Body esthetics	256	(41.0%)	129	(35.6%)	127	(48.3%)	0.001 *
Running technique	232	(37.1%)	121	(33.4%)	111	(42.2%)	0.025 *
Injury rehabilitation	78	(12.5%)	44	(12.2%)	34	(12.9%)	0.077
*p*-value		<0.001 ^#^		<0.001 ^#^		<0.001 ^#^	

* Significant difference compared by gender (*p* < 0.05); # Significant difference regarding the motive (*p* < 0.05).

## Data Availability

The collected data are included in this article and are the responsibility of the corresponding author. Some personal data of the participants cannot be provided due to confidentiality and respect for ethics.

## References

[1] Hulteen R.M., Smith J.J., Morgan P.J., Barnett L.M., Hallal P.C., Colyvas K., Lubans D.R. (2017). Global Participation in Sport and Leisure-Time Physical Activities: A Systematic Review and Meta-Analysis. Prev. Med..

[2] Vickers A.J., Vertosick E.A. (2016). An Empirical Study of Race Times in Recreational Endurance Runners. BMC Sports Sci. Med. Rehabil..

[3] Young B.A., Kniss J.R., Islas F. (2017). Lateral Foot Pain in a Recreational Runner. J. Orthop. Sports Phys. Ther..

[4] Decker G., Hunt D. (2019). Proximal Iliotibial Band Syndrome in a Runner: A Case Report. PM&R.

[5] Blagrove R.C., Brown N., Howatson G., Hayes P.R. (2020). Strength and Conditioning Habits of Competitive Distance Runners. J. Strength Cond. Res..

[6] García-Pinillos F., Soto-Hermoso V.M., Latorre-Román P.A. (2017). How Does High-Intensity Intermittent Training Affect Recreational Endurance Runners? Acute and Chronic Adaptations: A Systematic Review. J. Sport Health Sci..

[7] Llanos-Lagos C., Ramirez-Campillo R., Moran J., Sáez de Villarreal E. (2024). The Effect of Strength Training Methods on Middle-Distance and Long-Distance Runners’ Athletic Performance: A Systematic Review with Meta-Analysis. Sports Med..

[8] Llanos-Lagos C., Ramirez-Campillo R., Moran J., Sáez de Villarreal E. (2024). Effect of Strength Training Programs in Middle- and Long-Distance Runners’ Economy at Different Running Speeds: A Systematic Review with Meta-Analysis. Sports Med..

[9] Balsalobre-Fernández C., Santos-Concejero J., Grivas G.V. (2016). Effects of Strength Training on Running Economy in Highly Trained Runners: A Systematic Review with Meta-Analysis of Controlled Trials. J. Strength Cond. Res..

[10] Blagrove R.C., Howatson G., Hayes P.R., Hayes P.R. (2018). Effects of Strength Training on the Physiological Determinants of Middle- and Long-Distance Running Performance: A Systematic Review. Sports Med..

[11] Paavolainen L., Häkkinen K., Hämäläinen I., Nummela A., Rusko H. (1999). Explosive-Strength Training Improves 5-Km Running Time by Improving Running Economy and Muscle Power. J. Appl. Physiol..

[12] Spurrs R.W., Murphy A.J., Watsford M.L. (2003). The Effect of Plyometric Training on Distance Running Performance. Eur. J. Appl. Physiol..

[13] Turner A.M., Owings M., Schwane J.A. (2003). Improvement in Running Economy after 6 Weeks of PlyOmetric Training [Amelioration de l’Economie de Course Apres 6 Semaines d’Entrainement Pliometrique]. J. Strength Cond. Res..

[14] Yamamoto L.M., Lopez R.M., Klau J.F., Casa D.J., Kraemer W.J., Maresh C.M. (2008). The Effects of Resistance Training on Endurance Distance Running Performance among Highly Trained Runners: A Systematic Review. J. Strength Cond. Res..

[15] Walter S.D., Hart L.E., Sutton J.R., McIntosh J.M., Gauld M. (1988). Training Habits and Injury Experience in Distance Runners: Age-and Sex-Related Factors. Phys. Sportsmed..

[16] Alt T., Knicker A.J., Strüder H.K. (2020). Assessing Thigh Muscle Balance of Male Athletes with Special Emphasis on Eccentric Hamstring Strength. Phys. Sportsmed..

[17] García-Pinillos F., Lago-Fuentes C., Jaén-Carrillo D., Bujalance-Moreno P., Latorre-Román P.Á., Roche-Seruendo L.E., Ramirez-Campillo R. (2020). Strength Training Habits in Amateur Endurance Runners in Spain: Influence of Athletic Level. Int. J. Environ. Res. Public Health.

[18] Beattie K., Carson B.P., Lyons M., Rossiter A., Kenny I.C. (2017). The Effect of Strength Training on Performance Indicators in Distance Runners. J. Strength Cond. Res..

[19] Sullivan I.J., Johnson M.I., Hind K., Breen S., Francis P. (2019). Are Changes in Running Economy Associated with Changes in Performance in Runners? A Systematic Review and Meta-Analysis. J. Sports Sci..

[20] Rønnestad B.R., Mujika I. (2014). Optimizing Strength Training for Running and Cycling Endurance Performance: A Review. Scand. J. Med. Sci. Sport..

[21] Trowell D., Vicenzino B., Saunders N., Fox A., Bonacci J. (2020). Effect of Strength Training on Biomechanical and Neuromuscular Variables in Distance Runners: A Systematic Review and Meta-Analysis. Sports Med..

[22] Karp J.R. (2007). Training Characteristics of Qualifiers for the U.S. Olympic Marathon Trials. Int. J. Sports Physiol. Perform..

[23] Coluci M.Z.O., Alexandre N.M.C., Milani D. (2015). Construção de Instrumentos de Medida Na Área Da Saúde. Cienc. Saude Coletiva.

[24] Polit D.F., Beck C.T. (2006). The Content Validity Index: Are You Sure You Know What’s Being Reported? Critique and Recommendations. Res. Nurs. Health.

[25] Lynn M.R. (1986). Determination and Quantification of Content Validity. Nurs. Res..

[26] Polit D.F., Beck C.T., Owen S.V. (2007). Focus on Research Methods: Is the CVI an Acceptable Indicator of Content Validity? Appraisal and Recommendations. Res. Nurs. Health.

[27] Loudon J., Parkerson-Mitchell A. (2022). Training Habits and Injury Rate in Masters Female Runners. Int. J. Sports Phys. Ther..

[28] Griffin C., Egan B., Blake C., Horgan P. (2017). Training trends and injury incidences among irish distance runners. Br. J. Sports Med..

[29] Ramírez-Campilo R., Álvarez C., Henrríquez-Olguín C., Baez E.B., Martínez C., Andrae D.B., Izquierdo M. (2014). Effects of Plyometric Training on Endurance and Explosive Strength Performance in Competitive Middle- and Long-Distance Runners. J. Strength Cond. Res..

[30] García-Pinillos F., Lago-Fuentes C., Latorre-Román P.A., Pantoja-Vallejo A., Ramirez-Campillo R. (2020). Jump-Rope Training: Improved 3-Km Time-Trial Performance in Endurance Runners via Enhanced Lower-Limb Reactivity and Foot-Arch Stiffness. Int. J. Sports Physiol. Perform..

[31] Lauersen J.B., Bertelsen D.M., Andersen L.B. (2014). The Effectiveness of Exercise Interventions to Prevent Sports Injuries: A Systematic Review and Meta-Analysis of Randomised Controlled Trials. Br. J. Sports Med..

[32] Saragiotto B.T., Yamato T.P., Lopes A.D. (2014). What Do Recreational Runners Think about Risk Factors for Running Injuries? A Descriptive Study of Their Beliefs and Opinions. J. Orthop. Sports Phys. Ther..

[33] Duplanty A.A., Levitt D.E., Hill D.W., McFarlin B.K., Dimarco N.M., Vingren J.L. (2018). Resistance Training Is Associated with Higher Bone Mineral Density among Young Adult Male Distance Runners Independent of Physiological Factors. J. Strength Cond. Res..

[34] Luckin K.M., Badenhorst C.E., Cripps A.J., Landers G.J., Merrells R.J., Bulsara M.K., Hoyne G.F. (2018). Strength Training in Long-Distance Triathletes: Barriers and Characteristics. J. Strength Cond. Res..

[35] Denadai B.S., de Aguiar R.A., de Lima L.C.R., Greco C.C., Caputo F. (2017). Explosive Training and Heavy Weight Training Are Effective for Improving Running Economy in Endurance Athletes: A Systematic Review and Meta-Analysis. Sports Med..

[36] Trost S.G., Owen N., Bauman A.E., Sallis J.F., Brown W. (2002). Correlates of Adults’ Participation in Physical Activity: Review and Update. Med. Sci. Sports Exerc..

